# Evaluating Technology-Driven Strategies for Enhancing Patient Outreach for Preventive Care: A Systematic Review

**DOI:** 10.7759/cureus.79467

**Published:** 2025-02-22

**Authors:** Jincy Chacko, Kathleen Mazza, Kimon Stathakos, Doran Kim, Lorena Carlo

**Affiliations:** 1 Population Health Informatics, Northwell Health, New York City, USA; 2 Health Informatics, School of Health Professions, Rutgers University, Piscataway, USA; 3 Value Based Strategy Managed Care, Northwell Health, New York City, USA

**Keywords:** chatbots, health communication, preventive care, sms, technology-based outreach, value-based care

## Abstract

Preventive care is crucial in early disease detection and management, potentially reducing the need for extensive treatments and lowering healthcare costs. The integration of technology in health communication, especially through chatbots and SMS, offers innovative pathways to enhance patient adherence to recommended preventive screenings. However, evidence of the utility of these technologies outside immunization remains sparse. This systematic review aimed to evaluate the impacts of technology-based patient outreach methods, specifically chatbots and SMS, on response rates and care gap closures in preventive care screenings beyond immunizations. Utilizing the Patient/Population, Intervention, Comparison, Outcome (PICO) framework, this review searched five databases, including PubMed, CINAHL, Embase, IEEE Xplore, and ACM Digital Library, between January 2024 and July 2024. The search focused on studies evaluating the use of technology-based instruments like chatbots and mobile SMS, comparing them with traditional methods such as phone calls and mail. The primary outcomes investigated were response rates, failure to deliver, and the overall effectiveness of these technologies in the uptake of preventive screening measures. The search yielded 132 articles, with 10 meeting the inclusion criteria for full review. The findings highlight the significant potential of chatbots and SMS to enhance outreach programs for preventive care measure uptake, aligning with value-based care goals. However, the review highlighted the need for further methodologically robust research in this area that incorporates emerging technologies to strengthen conclusions. There is also a dearth of studies that evaluate emerging technologies like chatbots and address non-immunization preventative measures such as cancer screening and diabetes care. There is a noticeable gap in studies providing evidence on the cost-effectiveness of these technologies. While using chatbots and SMS in health communications appears promising, more comprehensive studies are required to understand their impact and cost-effectiveness in non-immunization screenings fully. Addressing these gaps is vital for developing scalable and sustainable preventive care strategies that can be integrated into the healthcare system.

## Introduction and background

Value-based care (VBC) is a well-established healthcare paradigm that has garnered heightened attention in the United States over the past decade due to enhancements in care delivery models facilitated by the Health Information Technology for Economic and Clinical Health (HITECH) Act [1]. VBC fundamentally shifts the focus of healthcare delivery from the reactive treatment of illnesses to the proactive prevention of diseases and the maintenance of wellness. This approach emphasizes the importance of primary and preventive care as defined by the Center for Medicare and Medicaid Services (CMS), advocating for prioritizing service quality over quantity, with performance often measured by patient health outcomes [2]. As the healthcare sector shifts towards a value-based care model with a stronger focus on cost management, preventive strategies - such as cancer screenings, medication adherence programs (e.g., aspirin for cardiovascular risk or statin therapy to reduce stroke or heart attack risk), and childhood immunization campaigns - are becoming increasingly essential to healthcare policies and practices [2,3]. The ability to preemptively identify health risks and intervene accordingly can significantly reduce the economic burden on healthcare systems by averting the need for complex and expensive treatments associated with advanced illnesses [4].

In recent years, multiple forms of preventive care, which include cancer screenings, medication adherence, and childhood immunizations, have seen a concerning decline, highlighting broader challenges in maintaining public health [2]. Despite a decrease in age-adjusted cancer rates over the past two decades, the incidence of cancer has continued to rise, paralleled by a 10% increase in cancer care costs from 2015 to 2020 due to an aging population and the introduction of expensive treatments [5]. Simultaneously, there has been a noticeable decline in the uptake of screenings, such as for colorectal and cervical cancer, which are critical for detecting early-stage cancers. This trend underscores the urgent need for targeted population health interventions to boost screening rates [5]. The 2021 National Health Interview Survey exposed significant challenges in medication adherence, where cost factors prevented 8.2% of adults aged 18-64 years from following prescribed medication regimens, highlighting the necessity for comprehensive strategies to enhance medication adherence as a preventive measure [6]. Additionally, childhood immunization rates have shown a troubling decline; coverage for essential vaccines like measles, mumps, and rubella (MMR), and others have failed to meet the Healthy People 2030 target by falling from 95% to 93% between 2019 and 2023. This decrease poses a significant measles infection risk for approximately 250000 kindergartners [7]. Collectively, these patterns of non-adherence across different preventive measures highlight the need for robust and integrated population health strategies that target adherence and equitably improve engagement with preventative care.

The promising role of technology in healthcare communication with patients presents the opportunity to enhance the engagement rates for preventive care [8]. Personalized health communication has been discussed for more than a decade [9]. However, its use with emerging technologies, such as artificial intelligence (AI) based chatbots, can tailor the outreach to individual preferences and needs while providing an interactive experience at a much lower cost than using human interventions. Systematic reviews focusing on the use of technology with preventative measures outcomes conducted by Mbuagbaw et al. and Pellowski and Kalichman have focused on immunizations and preventative treatment for HIV-positive populations and have tried to find a framework to generalize the findings from these studies [10,11]. Others, such as Crocker-Buqué et al. and Mekonnen et al. have reviewed studies on engagement and adherence with childhood vaccinations [12,13], while van Mierlo et al. have conducted a review of studies focusing on medication adherence [14]. These studies included the use of technology defined as "telehealth," "computerized interventions," or mobile/SMS and other traditional methods. None included the use of AI-based communication agents or chatbots. However, their findings suggest that a customized approach that tailors communication to a specific target population's characteristics and preferences is effective. This customized approach and potential avenue for cost-effectiveness are particularly crucial considering recent policy shifts that emphasize components of value-based healthcare, as discussed by Khullar et al. [15].

Despite advances, the literature shows a notable gap as follows: limited evidence on the effectiveness of chatbots and SMS for promoting preventive screenings beyond immunizations. While extensive reviews highlight traditional technologies like SMS, emails, and portal messages for boosting vaccination rates, their impact on screenings for other health conditions such as cancer, diabetes, and heart disease remains largely unexplored [10-14,16]. Given the significant health and financial burdens of these conditions, addressing this research gap is crucial. Moreover, technology adoption and healthcare delivery vary by region, making it essential to focus on synthesizing evidence from studies within the United States to develop strategies that address specific local barriers and needs. This systematic review seeks to bridge this knowledge gap by evaluating the impacts of technology-based outreach, specifically chatbots and SMS-on response rates and care gap closure for preventative care screenings. By doing so, it aimed to provide a comprehensive understanding of the utility of these technologies in a broader healthcare context, offering valuable insights for policymakers, healthcare providers, and patients alike.

## Review

Method

This review was guided by the Preferred Reporting Items for Systematic Reviews and Meta-Analyses (PRISMA) 2020 statement [17]. The Patient/Population, Intervention, Comparison, Outcome (PICO) framework which is a widely used tool for formulating focused research questions in a systematic review was used to develop search terms [18].

PICO Question

For patients eligible for preventive care screening (P), what is the impact of technology-based outreach interventions (I) such as SMS, email, automated/human telephone calls, and chatbots (C) on improving response rates and closing care gaps (O)? The components of the framework were derived from a query aimed at identifying scalable and cost-effective solutions to improve preventive screening rates. Focus was placed on preventative screening and outreach modalities, with a specific interest in new and emerging technologies such as chatbots used in healthcare communications. There was no restriction on the population during the search; therefore, it can be described as those eligible for preventative screenings. The intervention investigated was patient outreach using technology instruments such as chatbots, mobile SMS, email, and patient portal messages. Comparisons were sought with more traditional methods such as phone calls (automated or non-automated), postal mail, in-office, or no outreach exposures. The outcomes of interest included process measures such as response rate, failure to deliver, and effectiveness in preventative screening rate, sometimes also called compliance or completion rate.

Search Strategy

Databases searched encompassed a comprehensive range of sources suitable for health informatics research. These included PubMed, CINAHL, Embase, ACM Digital Library, and IEEE Xplore. Search terms were systematically identified to include all relevant aspects of the PICO framework. The key concepts derived from the PICO framework include value-based care, preventative screening, health promotion, gaps in care, health communication, chatbot, SMS, email, patient engagement, response rate, and gap closure. Additional terms were defined using specific MeSH terms, subject headings from databases such as PubMed and CINAHL, and additional synonyms and keywords found in preliminary search results. The comprehensive list of terms is provided in Table 1. The database search equations are provided in the table in the appendix.

**Table 1:** Comprehensive list of terms for search. PICO: Patient/Population, Intervention, Comparison, Outcome; HEDIS: Healthcare Effectiveness Data and Information Set

PICO	Key concepts	Mapped terms from PubMed and CINAHL
Population	Value-based care members	Primary prevention, value-based care, preventative healthcare, primary prevention, health promotion, preventive screening, population health, gaps in care, reimbursement, incentive, gaps in care, population health management, care gaps, health promotion, pay for performance, cancer screening, health maintenance, health screening, health plan employer data and information set, HEDIS, reimbursement, incentive
	Patients eligible for preventive screenings	
	Gaps in care	
Intervention	Communication	Health communication, outreach, communication, reminder systems, communication barrier
	Reminder	
Compare	Chatbot	Chatbot, natural language processing, dialog, bot, conversational agents, large language model
	Large language model	
	SMS	Text messaging, SMS, text messaging+, short messaging, smartphone, mobile messaging
	Text messaging	
Outcomes	Patient engagement and response	Health behavior, uptake, patient participation, response rate, patient engagement, patient compliance, close gaps, response rate, attitude to health, gaps close, compliance, behavior and behavior mechanisms, participation, gap closure

The primary search was conducted in PubMed in January 2024, which helped identify additional key terms. A Boolean operator was employed to refine the search, using AND to combine main concepts and OR to include synonyms and related terms. Search terms were expanded after the preliminary PubMed search. The most relevant results on the first page were reviewed for key terms within the article. Terms such as "conversational agents," "dialog," "reminder systems," and "smartphone" were added as synonyms for the outreach modality as well as "uptake" was added as part of the search terms for outcomes.

Embase was searched in February 2024 using a direct translation of the search in PubMed. CINAHL subject headings were searched for database-specific terms using MeSH synonyms and key concepts. CINAHL and IEEE Xplore were also searched in February 2024. After the initial search and review, a secondary search was conducted in July 2024, which included journal publications from the ACM digital library and the previously mentioned databases. The strategy was tailored to each database's specific syntax and search capabilities. No search filters were used in database searches due to an anticipated low body of literature and an apprehension of database filters excluding relevant studies. 

Screening Criteria

Studies were included based on the following criteria: they were published in English, peer-reviewed, and classified as original research. Eligible study designs encompassed randomized controlled trials, observational studies, comparative studies with concurrent controls, cohort studies, case-control studies, case series, qualitative studies, and mixed-method designs. Additionally, the studies had to evaluate the use of technology-based outreach tools, such as chatbots or mobile SMS, and report on patient engagement and/or completion rates for preventive screening.

Excluded study types were review articles or protocols only. Studies that were not accessible in full text or were focused on non-US-based populations were excluded. Studies with non-chatbot or SMS outreach interventions or those that do not report outcomes for patient engagement or gap closure were not included. Also, studies that evaluated outreach messages with a compulsory or mandated regime were excluded. Examples of such studies included employer-based outreach for interventions such as flu or COVID-19 during the emergent period of the pandemic. Such studies were deemed misaligned to the purpose of this review. Additional exclusion criteria were applied during the full-text review process for studies with inappropriate implementation.

Screening Process

EndNote 21, a reference management software, was used to aid the screening process for the literature search. Articles from all database searches were loaded into a single EndNote Library. Duplicates were identified and removed using automated identification within EndNote based on the Author, Year, and Title fields. Three co-authors (JC, DK, KS) independently reviewed the primary search results, and each article received a title and abstract review by at least two reviewers. Any disagreements were reconciled during deliberation, which helped clarify inclusion and exclusion criteria.

Records excluded at this stage were systematic reviews, research protocols, and studies that used interventions unrelated to chatbots or text messaging. For example, studies reported results for outreach via social media, which were excluded, considering the contended use of social media for health care purposes, unanimously by all reviewers. After the primary review, the records finalized for full-text review were tagged in EndNote, and an automated search was done within EndNote to find full-text for those articles. Any articles without full-text availability via EndNote were manually searched using databases and Google Scholar for full-text availability. Finally, one article was removed because only a conference abstract was available in peer-reviewed publication.

After the secondary screening, the new articles received a title and abstract review by three independent reviewers (JC, LC, KM) and all included articles received a full-text review by the same authors, ensuring that every article was reviewed by at least two reviewers. Disagreements were resolved through discussion to achieve consensus.

Data Extraction and Synthesis

Data extracted from the articles included the following: authors, publication year, title, language, keywords, main aim, journal name, study design, sample size, duration of study, age, gender, race, ethnicity, socioeconomic indicators, target population (health condition), outreach modality, duration of intervention, frequency of intervention, outcomes measured, statistical significance, effect sizes, limitations, author conclusions, future research, and recommendations.

The extracted data were systematically organized and stored in a tabular format using Microsoft Excel (Redmond, WA: Microsoft Corp.), allowing for structured analysis and visualization to assess homogeneity. However, significant methodological heterogeneity among the included studies precluded the possibility of conducting a meta-analysis. Notable variations were observed in key study characteristics, including differences in how study populations were defined and measured - such as assessing social vulnerability through language proficiency in one study, insurance coverage in another, and the area deprivation index in yet another. Additionally, inconsistencies in intervention design, including the type and frequency of preventive care, as well as variations in the duration between intervention implementation and outcome measurement, further contributed to this heterogeneity. Given these substantial differences, quantitative synthesis was not feasible. Instead, the findings are synthesized and interpreted using a narrative approach to provide a comprehensive and contextualized understanding of the results.

Quality Assessment

To assure the quality of the review and avoid the risk of bias, reviewer 1 (JC) and reviewer 2 (LC or KC) independently used the Mixed Methods Appraisal Tool (MMAT) version 2018 [19]. Conflicts of assessments were resolved by a third reviewer (LC or KM). This tool was deemed appropriate due to its accommodation in evaluating mixed study types. This assessment was not used to exclude any studies but only as an indicator of the quality of the reviewed studies.

Results

Study Selection

**Figure 1:**
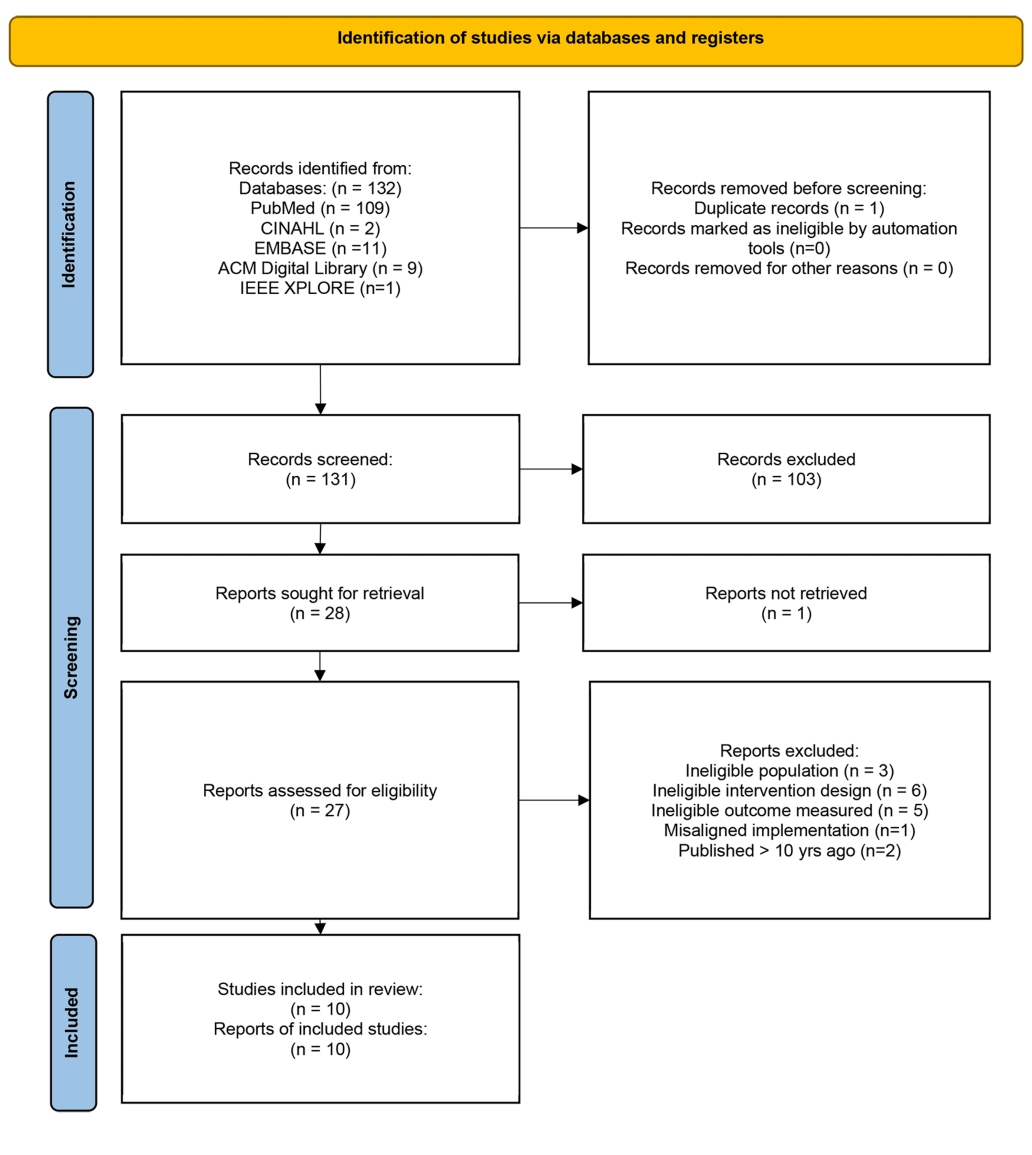
PRISMA flow diagram 2020. PRISMA: Preferred Reporting Items for Systematic Reviews and Meta-Analyses

The search results yielded 132 articles in total, of which the largest portion came from PubMed (109), followed by Embase (11), ACM Digital Library (9), CINAHL (2), and IEEE XPLORE (1). The initial screening process of removing one duplicate article and reviewing of title and abstracts for relevance resulted in 28 articles for inclusion. One study found to be relevant was not included because it was a conference abstract. The final full-text review of 27 articles resulted in 10 eligible articles for inclusion. The reasons for exclusion included non-US-based populations such as Canada or Australia which were not evident in the preliminary review, ineligible intervention design such as mixing technical intervention with in-person intervention for the same group within the study, or ineligible outcome measurement such as measurement of program success and acceptability rather than a report of process measures and preventative screening uptake. Additional exclusions were made for publications more than 10 years old and misaligned implementation where the intended study design was not followed in practice. Figure 1 and Table 2 present these results.

**Table 2:** Studies included in this review. HPV: human papillomavirus

References	Year	Title
Wagner et al. [20]	2021	Addressing logistical barriers to childhood vaccination using an automated reminder system and online resource intervention: a randomized controlled trial
Niederhauser et al. [21]	2015	Vaccines4Kids: assessing the impact of text message reminders on immunization rates in infants
Buttenheim et al. [22]	2022	Effects of ownership text message wording and reminders on receipt of an influenza vaccination: a randomized clinical trial
Lieu et al. [23]	2022	Effect of electronic and mail outreach from primary care physicians for COVID-19 vaccination of Black and Latino older adults: a randomized clinical trial
Wijesundara et al. [24]	2020	Electronic health record portal messages and interactive voice response calls to improve rates of early season influenza vaccination: randomized controlled trial
Mehta et al. [25]	2022	Effect of text messaging and behavioral interventions on COVID-19 vaccination uptake: a randomized clinical trial
Saccardo et al. [26]	2024	Field testing the transferability of behavioural science knowledge on promoting vaccinations
Fischer et al. [27]	2017	Appointment reminders by text message in a safety net health care system: a pragmatic investigation
Wang et al. [28]	2022	Initial experience with a COVID‑19 screening chatbot before radiology appointments
Keeshin and Feinberg [29]	2017	Text message reminder-recall to increase HPV immunization in young HIV-1-infected patients

Study Characteristics

**Figure 2:**
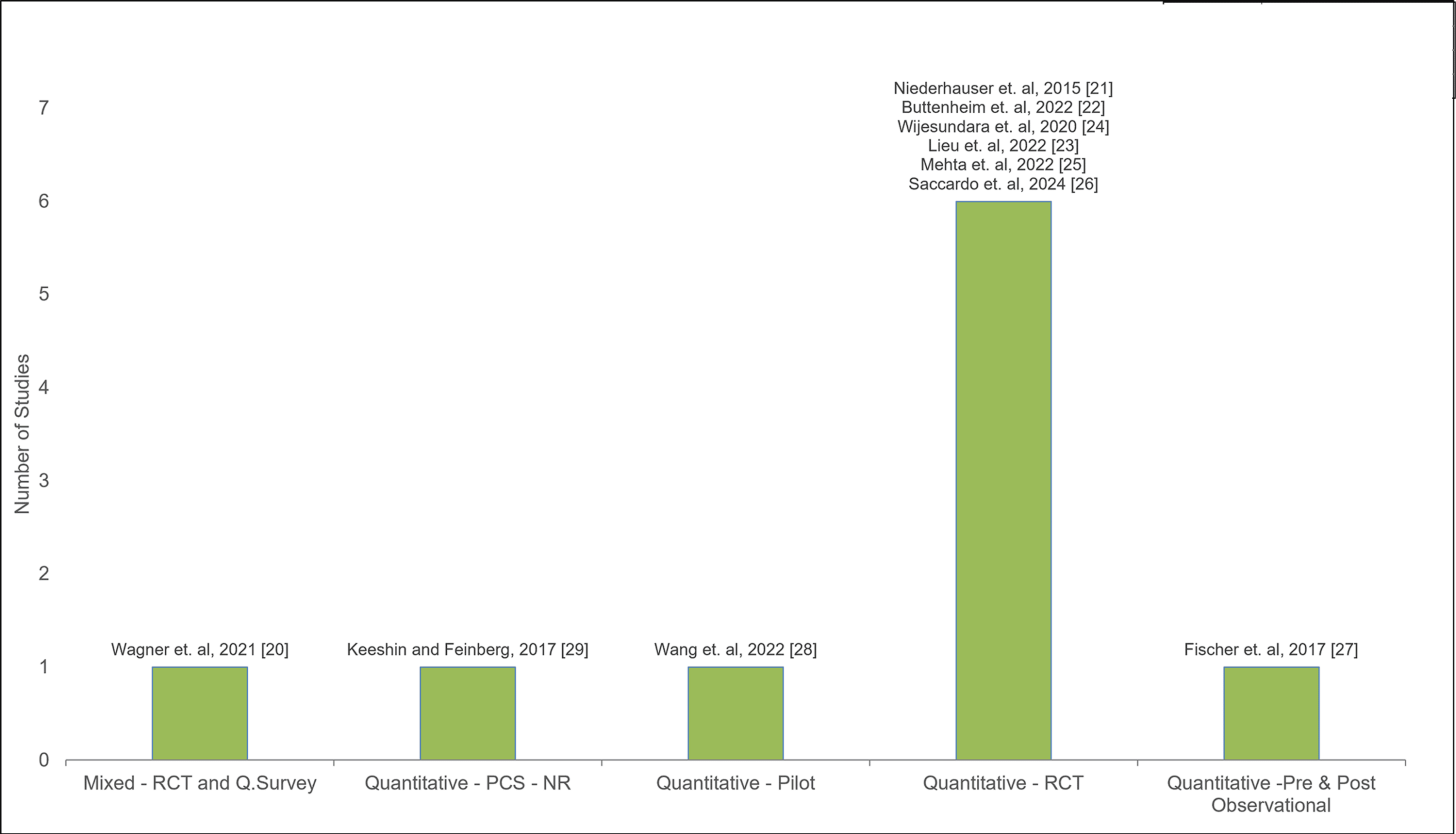
Distribution of study types included in the analysis, categorized by methodological approach. RCT: randomized control trial; PCS: prospective cohort study; NR: non-randomized; Q.Survey: qualitative survey

One out of 10 studies reviewed was a mixed-method study [20], with the remainder being quantitative, the largest subgroup of which was randomized controlled trials (RCTs) [21-26]. Figure 2 shows the count of studies by study type. Participant size for all studies ranged from 51 to 314824 participants, with the majority being conducted on mid-to-large population size (>4000). Nine out of 10 studies were conducted in the Western or Northeastern regions of the United States, representing urban or suburban populations [20-28]. Rural populations were included in the studies with smaller sample sizes, except for one large study from California which represented participants from all regions [23]. Geographical region and area classifications relative to population size in each study are presented in Table 3.

**Table 3:** Area classification and population size. F: false, the area was not represented well in the study population; T: true, the area was represented well in the study population

Index	Region of USA	Classification			Percentage of combined study participants	Study population size
		Urban	Suburban	Rural		
Niederhauser et al. [21]	West	F	T	T	0.01%	42
Wagner et al. [20]	West	F	T	T	0.05%	250
Keeshin and Feinberg [29]	Midwest	F	T	T	0.05%	255
Wang et al. [28]	West	F	T	T	0.86%	4687
Lieu et al. [23]	West	T	T	T	1.52%	8287
Buttenheim et al. [22]	Northeast	T	F	F	2.06%	11188
Mehta et al. [25]	Northeast	T	F	F	2.95%	16045
Fischer et al. [27]	West	T	F	F	16.71%	90895
Wijesundara et al. [24]	Northeast	T	T	F	17.94%	97607
Saccardo et al. [26]	West	T	F	F	57.86%	314824

**Figure 3:**
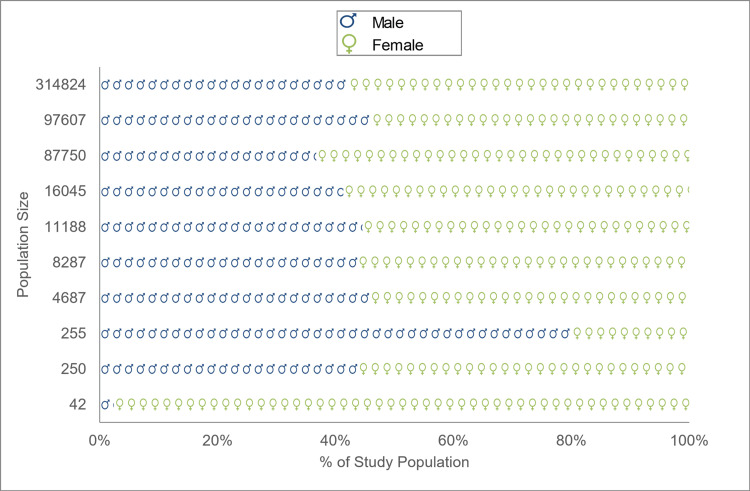
Gender distribution across study populations. The Y-axis represents the population size of each study, while the X-axis represents the percentage of the population reported as either male or female. There were no reports of non-binary gender.

The demographic characteristics most reported in all the studies included gender, race, and ethnic distribution. Three studies had skewed gender distribution [21,27,29]. One study had a larger Black population [29], while another specifically focused on and reported outcomes for minority populations categorized as Black or Latino [23]. Figure 3 and Table 4 display the gender and racial/ethnic distribution, respectively. Saccardo et al. and Wang et al. reported educational background [26,28]. Socioeconomic status (SES) was reported by six studies, differentially based on insurance type [20,25,29], marital status [21], or area deprivation index, making it difficult to synthesize a summary of SES for participants across the studies [23,26]. Fischer et al. recruited participants from a site that primarily caters to underserved and under-resourced populations [27].

**Table 4:** Racial and ethnic distribution. Note: Niederhauser et al. [21], Wijesundara et al. [24], and Wang et al. [28] did not explicitly report race and ethnicity, therefore are not included in this table. Saccardo et al. [26] mentioned multiple race groups but did not quantify all of them. NA: not available

References	Year	Asian Pacific Islander	White	Black	Multiracial	Other race	Unknown race	Hispanic	Non-Hispanic
Wagner et al. [20]	2021	0%	61.00%	4.00%	0.00%	11.00%	0.00%	15.00%	85.00%
Buttenheim et al. [22]	2022	2%	69.16%	20.26%	0.00%	3.46%	0.00%	4.86%	95.00%
Lieu et al. [23]	2022	0%	0.00%	29.40%	0.00%	0.00%	0.00%	45.60%	0.00%
Mehta et al. [25]	2022	8%	52.00%	29.30%	0.70%	4.10%	6.10%	6.00%	91.80%
Saccardo et al. [26]	2024	NA	48.76%	NA	NA	NA	NA	14.21%	85.79%
Fischer et al. [27]	2017	4%	25.70%	14.05%	0.00%	0.00%	19.50%	37.20%	62.80%
Keeshin and Feinberg [29]	2017	0%	25.50%	71.00%	0.00%	3.50%	0.00%	0.00%	0.00%

Intervention Designs and Outcome Measures

Eight out of 10 of the included studies focused on vaccination uptake [20-26,29], one study looked at primary care appointment adherence [27], and another focused on COVID-19 symptom screens [28]. The studies that focused on vaccinations vary in the type of vaccination and population in focus comprising childhood immunizations, human papillomavirus (HPV), influenza, and COVID-19 vaccinations. Seventy percent studied the impact of SMS [20-22,25-27,29], and only one utilized a chatbot for COVID-19 screen completion and measured subsequent vaccination rates for eligible participants [28]. Other electronic methods used in the studies included patient portal messages [23,24], emails [20,29], and interactive voice response [24]. Non-electronic methods included mailed letters and postcards for the comparison arm [23]. Mailed letters may have also been part of other studies as part of the comparison arm deemed "usual care" but were not explicitly reported.

Six studies performed multiple outreach activities ranging from two to four outreach attempts [20,21,23,25,27,29]. Uncontrolled exposure to other technology-based outreach attempts external to the study environment was reported in four studies [20,23,25,29]. Vaccination rate is the most reported outcome measure [20-26,28,29], followed by process measures [21-28], completion/compliance rate [22,24,25,27-29], and preferences for differential message text were commonly reported [22,23,25,26]. User experience was reported by only three studies [26-28]. The duration of the study intervention varied between two and 54 months without any indication of impact on study outcomes. Table 5 summarizes the methods and outcomes.

**Table 5:** Outreach methods, measures, and outcomes. T: method or measure was included in the design of the study; F: method or measure not included in the design of the study; IVR: interactive voice response

References	SMS	Chatbot	Portal messages	Email	Phone call	IVR	Postal mail or postcard	Uncontrolled exposure	Duration in months	Vaccination rate	Process measures	Compliance/completion rate	User experience	Effect of differential messaging	Barriers	Statistical significance (Y/N)	Variables impacting outcome
Wagner et al. [20]	T	F	F	T	T	F	F	T	54	T	F	F	F	F	F	N	NA
Niederhauser et al. [21]	T	F	F	F	F	F	F	T	7	T	T	F	F	F	T	N	NA
Mehta et al. [25]	T	F	F	F	T	F	F	T	2	T	T	T	F	T	F	N	NA
Buttenheim et al. [22]	T	F	F	F	F	F	F	F	6	T	T	T	F	T	F	Y	Race (White vs. Black)
Lieu et al. [23]	F	F	T	F	T	F	T	T	2	T	T	F	F	T	F	Y	Area deprivation index, race vs. ethnicity
Wijesundara et al. [24]	F	F	T	F	F	T	F	T	2	T	T	T	F	F	F	Y	Portal users vs. portal non-users
Saccardo et al. [26]	T	F	F	F	F	F	F	F	8	T	T	F	T	T	F	Y	Behaviorally targeted message variations
Fischer et al. [27]	T	F	F	F	F	F	F	F	18	F	T	T	T	F	F	Y	Standard appointment reminders
Wang et al. [28]	F	T	F	F	F	F	F	F	2	T	T	F	T	F	F	Y	Language preference (written)
Keeshin and Feinberg [29]	T	F	F	T	F	F	F	T	11	T	F	T	F	F	F	Y	None

Key Findings

Three interventional studies did not report significant outcomes for key measures of interest (completion rate, response rate, or vaccination rate) [20,21,25]. All three of these studies used SMS for the intervention arm but had variations in the comparison arm. They also reported uncontrolled exposure to additional technology-based outreaches.

Seven quantitative studies (70% of included studies) reported statistically significant results [22-24,26-29]. Wang et al. reported significant differences in vaccination rate and process outcomes, such as response rate, among participants with English as a preferred language [28]. Keeshin and Feinberg reported a significant increase in the uptake of HPV vaccination attributed to text message outreach compared to usual care [29]. Buttenheim et al. found racial influences in influenza vaccination uptake, showing that the white population responded positively to the “reserved vaccination message” while the black population responded slightly negatively to the same type of message, indicating variations in preferences among demographic groups [22]. Wijesundara et al. conducted a study between portal messaging impact and interactive voice recording outreach to demonstrate a significant difference in uptake and response rate of influenza vaccine by portal users [24]. Lieu et al. found that race, ethnicity, and area deprivation index were clear indicators for the response rate to differential messaging [23]. None of the above studies that looked for differences based on age or gender indicated any significant findings.

Risk of Bias Assessment

MMAT version 2018 assessment was used to assess methodological quality for all included studies [19]. Three studies were found to be methodologically sound - a mixed-method study with an RCT arm and two non-randomized studies [20,27,29]. Wang et al. indicated a higher risk of non-response bias at 42% [28]. The study by Keeshin and Feinberg reported uncontrolled exposure among the control group, which was addressed during analysis and, therefore, did not detract from the quality of the outcomes [29]. This review included six RCTs, of which only Saccardo et al. indicated the blinded quality of the research analysis [26]. Two studies reported clearly that the analysis was not blinded [22,24], and the other three studies did not report sufficient information to conclude [21,23,25]. The risk of non-response bias was prevalent among the RCT studies, ranging from 32% to 42% for studies that reported the number [23,25]. Only Buttenheim et al. reported an acceptable non-response rate of 8% for the intervention group and 10% for the control group [22]. Overall, the RCT study by Niederhauser et al. had the most limiting quality factors, which included incomparable baseline population characteristics, among other limitations mentioned above [21]. Table 6 includes the visual results of the quality assessment [19].

**Table 6:** Risk of bias assessment: mixed methods appraisal tool version 2018. Y: "Yes," study meets quality assessment requirements; C: "Can't tell," unsure if the study meets requirements based on provided data in the publication; N: "No," study does not meet the quality assessment requirements; MMAT: Mixed Methods Appraisal Tool

References	Year	MMAT 2018 study design category applied	Q1	Q2	Q3	Q4	Q5
Wagner et al. [20]	2021	Mixed methods studies (includes 1 and 2)	Y	Y	Y	Y	Y
Wang et al. [28]	2022	Quantitative descriptive studies	Y	Y	Y	N	Y
Keeshin and Feinberg [29]	2017	Non-randomized studies	Y	Y	Y	Y	Y
Niederhauser et al. [21]	2015	Randomized controlled trials	Y	N	C	C	Y
Buttenheim et al. [22]	2022	Randomized controlled trials	Y	Y	Y	N	Y
Wijesundara et al. [24]	2020	Randomized controlled trials	Y	Y	N	N	Y
Lieu et al. [23]	2022	Randomized controlled trials	Y	Y	N	C	Y
Mehta et al. [25]	2022	Randomized controlled trials	Y	Y	N	C	Y
Saccardo et al. [26]	2024	Randomized controlled trials	Y	Y	Y	Y	Y
Fischer et al. [27]	2017	Non-randomized studies	Y	Y	Y	Y	Y

Discussion

The systematic review presented in this study aimed to assess the literature for evidence of the effectiveness of technology-based outreach methods for increasing preventative screening rates compared to traditional methods. This discussion synthesizes the results, examines the key findings and their implications for health informatics and population health management, and suggests directions for future research.

Study Areas

There has been increased focus on SMS-based interventions, with a few studies also incorporating portal messages as a modality and chatbots having received scarce attention to date in outreach for preventative care. Email is now considered a part of the traditional mode of communication, along with interactive voice response (IVR) [24], postal mail or postcards [23,25], and phone calls by humans [20,23,25]. The studies have varied demographics, with the larger studies incorporating a mixed population regarding race and ethnicity with some focus on SES. However, each study varied in the variable used to determine SES (Medicaid vs. non-Medicaid, education level, income level, or area deprivation index), reflecting a lack of standardization in this area.

Some of the included studies assessed behavioral impact using message variation, which resulted in success, especially in denoting a preference between minority vs. non-minority groups and resourced vs. under-resourced groups [22,23,25,26]. The varied message types tested included strategies to convey "ownership" of a vaccination vial, such as using terms like "reserved for you," as well as efforts to increase trust through a primary care physician's endorsement or by providing detailed information about the immunization. This indicates a need to personalize messages based on population or sub-population characteristics when engaging in mass messaging technologies such as SMS and chatbots.

Most of the research is on outreach for vaccination (adult or child) and appointment adherence, which can eventually lead to other preventative screening goals. Other screens, such as early cancer detection or medication adherence, have had no focus. Cancer screenings, including mammography, colorectal cancer screening, and cervical cancer testing, are essential preventive measures that have demonstrated substantial reductions in mortality when implemented effectively [30]. The lack of emphasis on these screenings in existing studies raises concerns about gaps in the research agenda, potentially reflecting biases in funding priorities. Additionally, cancer detection often requires more resources, including diagnostic equipment, follow-up procedures, and long-term outcome tracking, making them less attractive for research compared to vaccinations, which are frequently perceived as a one-time intervention with clear and immediate benefits, mainly when funding and resources are limited [31].

Effectiveness of Technology-Based Outreach

The findings of this systematic review highlight a complex and nuanced landscape regarding the effectiveness of technology-driven strategies for enhancing preventive screening outreach. The studies included in this review demonstrate both consistencies and contradictions, emphasizing the need to carefully interpret the results in light of methodological quality, study design, and demographic influences.

Among the included RCTs, the gold standard of quality research, three studies looked at COVID-19 vaccination rates and studied specific message variations [23,25,26]. Of these, the study by Saccardo et al. was of the highest quality and demonstrated a significant difference in response to differential messaging, especially those with "ownership" or "endorsed by primary care provider (PCP)" text for COVID booster uptake [26]. This study further highlighted a discrepancy between perceived behaviors, as indicated by previous survey results, which suggested that participants would be more inclined to vaccinate if vaccinations were bundled, and the practical outcome, where participants were actually more inclined when presented with messages emphasizing ownership and primary care provider (PCP) endorsement.

Lieu et al. studied the impact of cultural variation in messages for COVID-19 uptake and found comparable results using portal messages [23]. However, Mehta et al. contradicted these findings [25]. It is important to note that the latter two studies only lacked the blinded nature of the RCT and acknowledged the presence of uncontrolled exposure, likely influenced by widespread global efforts to mitigate the pandemic during that period. Additionally, missing or documented high rates of non-response, limit the completeness of the data and raise concerns regarding the reliability and generalizability of the findings.

Buttenheim et al. and Wijesundara et al. also conducted RCT studies with moderate quality concerns that studied influenza vaccine rates [22,24]. Both concluded that there was a statistically significant difference in the intervention group, with the former presenting results in support of differential messaging resulting in better compliance (reserved vs. standard message) and the latter concluding that portal messages were more impactful than IVRs. It is important, however, to note that portal users may not be comparable at baseline as there could be accessibility issues, which were not explored in the study. Additionally, a study by Wijesundara et al. reported a response rate of 51.9%, raising concerns about non-response bias [24]. Compounding on quality concerns, both studies acknowledged the influence of the COVID-19 pandemic on vaccination behavior, leading us to believe that this area would benefit from further research to corroborate the findings.

Two studies with RCT arms examining the use of SMS-based interventions for childhood vaccinations reported no statistically significant differences between comparison groups [20,21]. Between the two studies, Wagner et al. have a methodologically sound RCT arm and measured the percentage of days under-vaccinated [20]. They highlighted logistical barriers such as transportation and lack of knowledge secondary to decreased utilization among the Medicaid populations as potential blockers to outreach response. The study by Niederhauser et al. had significantly more limitations, with a smaller sample size of 42, a higher dropout rate, and a higher rate of reported barriers among the intervention arm, leading to the inability to generalize the findings [21].

The studies by Fischer et al. and Keeshin and Feinberg were characterized as non-RCTs as per the MMAT 2018 definitions [27,29], studying the impact of outreach using SMS on HPV vaccination rates and appointment compliance rates, respectively [19]. Both reported statistically significant results for the intervention group, which had higher rates of vaccination or kept appointments. Wang et al. being the only study that assessed the impact of chatbot technology in the preventive screening of COVID-19 and subsequent vaccination for eligible participants, was a quantitative descriptive study that can best be characterized as a positive step in the direction of testing chatbots for mass communication [28]. The response rate from chatbots was like that of other studies using SMS, and the authors claim a significant difference among the intervention group. The authors reported that the written language preference of "English" was an influencing factor in compliance, indicating the importance of incorporating language preferences when performing outreach using mass communication technology. The statistically significant findings of these studies are promising but require more research with a robust design to corroborate the findings and apply them to broader practice.

Risk of Bias and Study Limitations

While the included studies demonstrate promising outcomes, bias-related limitations warrant consideration when interpreting their findings. Measurement bias emerged as a key concern, as many studies relied on self-reported outcomes or electronic health records, which may have led to underreporting or incomplete data capture. Selection bias was also evident, with participants who had higher digital literacy and better access to technology being more likely to respond to digital interventions. For example, studies involving portal messaging systems often included participants who were already more engaged with healthcare than non-users, introducing a selection bias that limited generalizability to broader populations. Wagner et al. further highlighted disparities caused by pre-existing barriers, which disproportionately affected the intervention group and were not accounted for prior to randomization [20].

Studies also reported the influence of confounding variables, such as public health campaigns and the pandemic context, which may have independently affected outcomes and response rates. Additionally, inconsistencies in subgroup analyses posed challenges for evaluating intervention effectiveness across diverse demographics. For instance, while most studies accounted for race/ethnicity, few examined age groups, and socioeconomic status (SES) definitions varied widely, limiting insights into equity gaps and population-specific needs.

Specific randomized controlled trials (RCTs) also revealed weaknesses in group comparability and participant adherence, which are critical for isolating intervention effects. The study by Niederhauser et al. reported a higher proportion of married participants in the intervention group (58% vs. 30%), which could potentially influence parental adherence to childhood vaccination schedules, as single caregivers may face more barriers [21]. Similarly, Wijesundara et al. in their study identified imbalances between intervention arms, with portal users (older, female, and high utilizers) resembling the control group, whereas the interactive voice response (IVR) group differed significantly in demographics [24]. These discrepancies may explain why portal users were more likely to complete vaccinations, highlighting the role of baseline differences rather than the intervention itself. While several studies exhibited methodological rigor, others underscore the need for more robust designs that minimize bias and improve generalizability.

Gaps Identified and Future Research

The current review highlights a predominant focus on immunizations within the studies of technological interventions, particularly for preventive health screenings. However, there is a notable gap in research on using technology to enhance adherence to screenings for non-communicable diseases such as cancer, where early detection is vital for outcomes. Exploring how digital interventions might influence behaviors related to preventive screenings for chronic diseases is a crucial area for further study.

Another gap relates to the influence of SES and healthcare accessibility on the efficacy of technology-based interventions. Many studies failed to analyze the role of economic disparities, digital literacy, and language barriers in shaping intervention outcomes. Research should begin incorporating more standardized metrics for SES, such as a social vulnerability index provided by the Centers for Disease Control and Prevention (CDC) that includes several key social and environmental factors and enables a relative comparison within a population [32]. Only one study reported multi-lingual (English and Spanish) messaging [27], while some did discuss the possibility of the language barrier being a confounding factor [28]. Others are assumed to be in English as there was a lack of reporting of the language of the messages used. Future studies should attempt the incorporation of translation services as part of the technology implementation. Addressing these elements is vital for achieving true equity in the healthcare industry. A further gap is the cost-effectiveness of these interventions. Few studies provided data on whether these approaches are economically viable or lead to reduced healthcare costs over time. Evaluating the financial sustainability of interventions is essential for determining their scalability and potential for widespread implementation.

Emerging technologies, such as AI-based chatbots, also remain underexplored. While one study demonstrated the feasibility of chatbot-enabled outreach for COVID-19 screenings and vaccinations, there is little comparison with SMS reminders. Given the rise of artificial intelligence and machine learning in health communications, future studies should examine their ability to customize messages and optimize engagement. Evidence from behavioral science-based messaging strategies suggests that personalized content can improve response rates, underscoring the need to evaluate these technologies for real-world effectiveness [22,23,26].

## Conclusions

This systematic review evaluated the available evidence for the effectiveness of technology-based outreach strategies, including SMS and chatbots, in improving uptake of preventative measures. The findings indicate that these interventions can improve response rates and close care gaps. However, critical gaps and methodological limitations exist in the current literature, including selection biases and generalizability issues, raising concerns about equitable access. Behavioral science-informed approaches, such as personalized and culturally tailored messaging, showed some promise but lacked consistent results across different studies, highlighting the need for continued research. Emerging tools, such as chatbots, offer more personalized health communication opportunities and remain understudied at the time of this review.

Beyond immunizations, the review underscores the need for research on digital interventions for other preventive screenings, such as cancer and diabetes. Few studies examined the cost-effectiveness of these strategies, leaving questions about their economic sustainability and scalability. Future research should incorporate cost analyses and longitudinal designs to evaluate preventative methods with varied outcome measurement lengths. Technology-based efficiencies warrant further studies to advance population health strategies as healthcare gears to address equity and scalability issues and look to foster a more inclusive and cost-effective approach to preventive care.

## Appendices

**Table 7:** Database search equations.

Database	Equation
PubMed	("Primary prevention"[MeSH Terms] OR "Health promotion"[MeSH Terms] OR "reimbursement, incentive"[MeSH Terms] OR "Value-Based Care"[Title/Abstract] OR "Preventive screening"[Title/Abstract] OR "Gaps in Care"[Title/Abstract] OR "Care gaps"[Title/Abstract] OR "Pay for Performance"[Title/Abstract] OR "Health Maintenance"[Title/Abstract] OR "HEDIS"[Title/Abstract]) AND ("Health communication"[MeSH Terms] OR "Reminder Systems"[MeSH Terms] OR "Chatbot"[Title/Abstract] OR "Dialog"[Title/Abstract] OR "Conversational Agents"[Title/Abstract] OR "Large language model"[Title/Abstract] OR "Outreach"[Title/Abstract]) AND ("Text messaging"[MeSH Terms] OR "Electronic mail"[MeSH Terms] OR "SMS"[Title/Abstract] OR "Short messaging"[Title/Abstract] OR "Mobile messaging"[Title/Abstract] OR "Smartphone"[Title/Abstract]) AND ("Health behavior"[MeSH Terms] OR "Uptake"[Text Word] OR "Patient engagement"[Text Word] OR "Response rate"[Text Word] OR "Compliance"[Text Word] OR "Participation"[Text Word] OR "Close Gaps"[Text Word] OR "Gap closure"[Text Word])
Embase	('primary prevention'/exp/mj OR 'health promotion'/exp/mj OR 'reimbursement'/exp/mj OR 'value-based care':ti,ab,kw OR 'preventive screening':ti,ab,kw OR 'gaps in care':ti,ab,kw OR 'care gaps':ti,ab,kw OR 'pay for performance':ti,ab,kw OR 'health maintenance':ti,ab,kw OR 'hedis':ti,ab,kw) AND ('medical information'/exp/mj OR 'reminder system'/exp/mj OR 'chatbot':ti,ab,kw OR 'dialog':ti,ab,kw OR 'conversational agents':ti,ab,kw OR 'large language model':ti,ab,kw OR 'outreach':ti,ab,kw) AND ('text messaging'/exp/mj OR 'e-mail'/exp/mj OR 'sms':ti,ab,kw OR 'short messaging':ti,ab,kw OR 'mobile messaging':ti,ab,kw OR 'smartphone':ti,ab,kw) AND ('health behavior'/exp OR 'health behavior' OR 'uptake':ti,ab,kw,de,dn,df,mn,tn OR 'patient engagement':ti,ab,kw,de,dn,df,mn,tn OR 'response rate':ti,ab,kw,de,dn,df,mn,tn OR 'compliance':ti,ab,kw,de,dn,df,mn,tn OR 'participation':ti,ab,kw,de,dn,df,mn,tn OR 'close gaps':ti,ab,kw,de,dn,df,mn,tn OR 'gap closure':ti,ab,kw,de,dn,df,mn,tn)
CINAHL	( ((MH "Preventive Health Care") OR "primary prevention" OR (MM "Population Health") OR (MM "Population Health Management") OR (MM "Health Promotion") OR (MM "Reimbursement, Incentive") OR (MM "Cancer Screening") OR (MM "Health Screening") OR (MM "Health Plan Employer Data and Information Set") OR "gaps in care") ) AND ( ("chatbot" OR "bot" OR "Conversational Agents" OR (MM "Natural Language Processing") OR "Outreach" OR "Large Language Model") ) AND ( (MM "Text Messaging+") OR (MM "Email") OR (MM "Smartphone") OR "E-mail" OR "Short message" OR "SMS”) ) AND ( (MH "Patient Participation") OR (MH "Patient Compliance") OR (MH "Attitude to Health") OR (MH "Behavior and Behavior Mechanisms") OR "Response Rate" OR "Close Gaps" OR "Gaps Close" )
IEEE Xplore	(("Title": "Primary prevention" OR "Abstract": "Primary prevention") OR ("Title": "Health promotion" OR "Abstract": "Health promotion") OR ("Title": "reimbursement, incentive" OR "Abstract": "reimbursement, incentive") OR ("Title": "Value-Based Care" OR "Abstract": "Value-Based Care") OR ("Title": "Preventive screening" OR "Abstract": "Preventive screening") OR ("Title": "Gaps in Care" OR "Abstract": "Gaps in Care") OR ("Title": "Care gaps" OR "Abstract": "Care gaps") OR ("Title": "Pay for Performance" OR "Abstract": "Pay for Performance") OR ("Title": "Health Maintenance" OR "Abstract": "Health Maintenance") OR ("Title": "HEDIS" OR "Abstract": "HEDIS") OR ("Title": "Preventative Health Care" OR "Abstract": "Preventative Health Care") OR ("Title": "Population Health" OR "Abstract": "Population Health") OR ("Title": "Population Health Management" OR "Abstract": "Population Health Management") OR ("Title": "Cancer Screening" OR "Abstract": "Cancer Screening") OR ("Title": "Health Screening" OR "Abstract": "Health Screening") OR ("Title": "Health Plan Employer Data and Information Set" OR "Abstract": "Health Plan Employer Data and Information Set")) AND (("Title": "Health communication" OR "Abstract": "Health communication") OR ("Title": "Reminder Systems" OR "Abstract": "Reminder Systems") OR ("Title": "Outreach" OR "Abstract": "Outreach") OR ("Title": "Communication" OR "Abstract": "Communication") OR ("Title": "Communication barrier" OR "Abstract": "Communication barrier")) AND (("Title": "SMS" OR "Abstract": "SMS") OR ("Title": "Short messaging" OR "Abstract": "Short messaging") OR ("Title": "Mobile messaging" OR "Abstract": "Mobile messaging") OR ("Title": "Smartphone" OR "Abstract": "Smartphone") OR ("Title": "Natural Language Processing" OR "Abstract": "Natural Language Processing") OR ("Title": "Text messaging" OR "Abstract": "Text messaging") OR ("Title": "Chatbot" OR "Abstract": "Chatbot") OR ("Title": "Dialog" OR "Abstract": "Dialog") OR ("Title": "Conversational Agents" OR "Abstract": "Conversational Agents") OR ("Title": "Large language model" OR "Abstract": "Large language model")) AND (("Title": "Health behavior" OR "Abstract": "Health behavior") OR ("Title": "Uptake" OR "Abstract": "Uptake") OR ("Title": "Patient engagement" OR "Abstract": "Patient engagement") OR ("Title": "Response rate" OR "Abstract": "Response rate") OR ("Title": "Compliance" OR "Abstract": "Compliance") OR ("Title": "Participation" OR "Abstract": "Participation") OR ("Title": "Close Gaps" OR "Abstract": "Close Gaps") OR ("Title": "Gap closure" OR "Abstract": "Gap closure") OR ("Title": "Patient Participation" OR "Abstract": "Patient Participation") OR ("Title": "Patient Compliance" OR "Abstract": "Patient Compliance") OR ("Title": "Attitude to Health" OR "Abstract": "Attitude to Health") OR ("Title": "Behavior and Behavior Mechanisms" OR "Abstract": "Behavior and Behavior Mechanisms") OR ("Title": "Response Rate" OR "Abstract": "Response Rate") OR ("Title": "Close Gaps" OR "Abstract": "Close Gaps") OR ("Title": "Gaps Close" OR "Abstract": "Gaps Close"))
ACM Digital Library	"Primary prevention" OR "Health promotion" OR "reimbursement, incentive" OR "Value-Based Care" OR "Preventive screening" OR "Gaps in Care" OR "Care gaps" OR "Pay for Performance" OR "Health Maintenance" OR "HEDIS" OR "Preventative Health Care" OR "Population Health" OR "Population Health Management" OR "Cancer Screening" OR "Health Screening" OR "Health Plan Employer Data and Information Set" AND "Health communication" OR "Reminder Systems" OR "Outreach" OR "Communication" OR "Communication barrier" OR "SMS" OR "Short messaging" OR "Mobile messaging" OR "Smartphone" OR "Text messaging" OR "Chatbot" OR "Dialog" OR "Conversational Agents" OR "Large language model" AND "Health behavior" OR "Uptake" OR "Patient engagement" OR "Response rate" OR "Compliance" OR "Participation" OR "Close Gaps" OR "Gap closure" OR "Patient Participation" OR "Patient Compliance" OR "Attitude to Health" OR "Behavior and Behavior Mechanisms" OR "Response Rate" OR "Close Gaps" OR "Gaps Close"
